# Guns in rosettes: The Arabidopsis chemical weapons arsenal

**DOI:** 10.1093/plphys/kiaf411

**Published:** 2025-09-23

**Authors:** Marc Somssich, Daniel J Kliebenstein, Tonni Grube Andersen

**Affiliations:** Max Planck Institute for Plant Breeding Research, Cologne, NW 50829, Germany; Cluster of Excellence on Plant Sciences (CEPLAS), Cologne, NW 50829, Germany; Department of Plant Sciences, University of California, Davis, CA 95616, USA; Plant Biology Graduate Group, University of California, Davis, CA 95616, USA; Max Planck Institute for Plant Breeding Research, Cologne, NW 50829, Germany; Cluster of Excellence on Plant Sciences (CEPLAS), Cologne, NW 50829, Germany

## Abstract

*Arabidopsis thaliana* (hereafter Arabidopsis) is a small plant with a fast generation time and a well-annotated genome, which makes it ideal for research labs. It is arguably the most used model species in basic plant sciences. Over the past half century, studies in Arabidopsis have generated enormous insight into fundamental principles of plant life, ranging from mechanistic molecular biology to the complexities of interacting ecosystems. Based on research in Arabidopsis, we now understand that while basic cellular metabolism is generally conserved across species, variation in specialized metabolite enzymes gives rise to complex bouquets of chemical weapons that are tightly interwoven with the environment. Understanding how these are produced, regulated, and—especially—how they are deployed remains a key research area for plant immunity. The breadth of work in Arabidopsis provides a unique window into this complicated aspect of life as a plant. We are happy to have an opportunity to share our common interest in these aspects in this review. Due to space constraints, we focus on compounds produced by Arabidopsis with demonstrated antimicrobial properties. We hope that this focus (despite our eagerness to write more) will inspire new avenues of research that will contribute to a more complete understanding of immunity.

## Teaser

Arabidopsis remains one of the best-studied models today and has a number of remarkable chemical defense systems which actively engage, interact, and prevent a plethora of enemies from attacking it.

Advances box

- Arabidopsis is one of the most used model species and contains a number of chemical defense systems with distinct functions, which have formed the basis for our current understanding of plant immunity.
- Chemical defense systems are the “weapons” plants deploy in their fight against enemies, but our knowledge of how and against what they work is limited.
- Arabidopsis primarily uses glucosinolates, phenylpropanoids, and terpenoids as defenses; however, how these molecular strategies are coordinated across tissues and throughout the plant's lifespan is unknown.
- We provide an overview of our current knowledge on Arabidopsis and its defense systems in the context of microbial battlefronts, as well as suggestions for future research directions.

## Introduction

*Arabidopsis thaliana* (hereafter referred to as Arabidopsis) is a small plant with a fast generation time and a well-annotated genome, making it ideal for research laboratories. It is arguably the most commonly used model species in basic plant sciences (Somssich 2022). Over the past half century, studies in Arabidopsis have generated enormous insight into fundamental principles of plant life, ranging from mechanistic molecular biology to the complexities of interacting ecosystems (Provart et al. 2016). Based on research in Arabidopsis, we now understand that while basic cellular metabolism is generally conserved across species, variation in specialized metabolite enzymes gives rise to complex assortments of chemical weapons that are tightly interwoven with the environment. Understanding how these chemicals are produced, regulated, and—especially—deployed remains a key research area for plant immunity. The breadth of work in Arabidopsis provides a unique window into this complex aspect of life as a plant. We are happy to have the opportunity to share our common interest in these aspects in this review. Due to space constraints, we focus on compounds produced by Arabidopsis that have demonstrated *antimicrobial* properties. We hope that this focus (despite our eagerness to write more) will inspire new avenues of research that will contribute to a more complete understanding of immunity.

### Chemical defenses in plants: finding the right words

Like any living organism, plants continually produce compounds crucial for growth. The driving force behind these metabolic processes is photosynthesis, which allows plants to produce an astounding array of metabolites with specialized roles. In their essence, these compounds protect the ability to photosynthesize by killing organisms that are interested in devouring or in other foul ways harming the plant. These metabolites, which typically originate from precursors obtained from general biosynthesis pathways, are not directly involved in cellular viability functions but serve specialized roles in protection against environmental challenges. Historically, the functions of these metabolites have confused and frustrated researchers to great extents. Metabolites of such a class have switched between being considered defense-related or waste products from enzymes associated with well-described biochemical cycles. One can almost imagine the heat of the underlying discussions. For consensus, or peace, they were termed secondary—or specialized—compounds versus the general, or primary metabolic compounds (Hartmann 2007; Pichersky and Lewinsohn 2011; Erb and Kliebenstein 2020). However, with our current-day molecular insights, it is now clear that they are vital for protection via, for example, chemical defense systems (Erb and Kliebenstein 2020).

These specialized defense metabolites have traditionally been categorized into *phytoanticipins* and *phytoalexins*, based on when and how the plant initiates and deploys their functions. *Phytoanticipins* are low-molecular-weight defense compounds that are either already present in plants before infection or are produced in response to an infection from preexisting precursors (Van Etten et al. 1994). On the other hand, *phytoalexins* are defined as low-molecular-weight defense compounds that are synthesized and accumulated de novo after infection (Van Etten et al. 1994). Recently, a third category termed *phytoavengins* was proposed. These have been defined as low-molecular-weight defense agents that are enzymatically activated from inactive preexisting constituents after plant tissue damage, thereby providing further distinction between defense compounds that are produced and stored in their active (phytoanticipin) or inactive (phytoavengin) forms (Kliebenstein and Kvitko 2023).

As our understanding deepens, the traditional definitions of terms such as “secondary” or “specialized” metabolites, as well as phytoanticipin, phytoalexin, and phytoavengin, become increasingly blurred. This reflects more nuanced insights into the interconnection of these metabolites with general processes and a broader role in plant life (Erb and Kliebenstein 2020; Lou et al. 2022; Kliebenstein and Kvitko 2023). In this review, we refer to the discussed compounds collectively as *defense compounds*, as we focus on their function as chemical weapons.

### How do plants know when to defend?

Similar to humans and their adaptive immune systems, plants have strategies for dealing with microbial threats. To monitor their surroundings, they employ transmembrane receptor-like kinases (RLKs) to constantly eavesdrop on their extracellular environment via sensor domains that protrude from the cell into the apoplast (Ngou et al. 2022; Huang and Joosten 2025). Microbes carry distinct molecular fingerprints—known as microbe-associated molecular patterns—which, upon recognition in the apoplast by the RLKs, trigger a process called pattern-triggered immunity (PTI; Jones et al. 2024). PTI is initiated by relaying information of microbial presence through an intracellular phosphorelay, which leads to a burst of reactive oxygen species, deposition of callose at the plasmodesmata, and/or the deployment of chemical weapons. All relatively polite preventive measures that inform invasive microbes that they are not welcome.

But pathogenic microbes are in for the fight, and so a molecular arms race occurs between plants and microbes. One of the most prominent tactics that microbes deploy to evade extracellular detection is the release of effector proteins into the plant cell, which, if successful, modulate the plant cell and sabotage the PTI-triggered immune responses (Jones et al. 2024). This creates a vulnerability in the plant's defenses that the microbe can exploit to achieve successful infection. To counter this, plants have, in turn, evolved intracellular receptors [often of a type called nucleotide-binding leucine-rich repeat (NLR) receptors], which are designed to either detect the microbial effector proteins or the changes the effector causes in the plant cell (Jones et al. 2024). Detection of an effector, or effector activity, triggers a second form of immune response called effector-triggered immunity (ETI). If the PTI can be considered the plant's “warning shot,” then ETI is the plant taking the attack seriously.

ETI and PTI overlap in large parts and can amplify each other (Ngou et al. 2021; Yuan et al. 2021). When triggered in the same cell, the outcome can be downright deadly since the plant has not only detected a microbe in its environment (which can be pathogenic but also beneficial) but now also has actual evidence that its defenses have been actively breached (Jones et al. 2024). For this event, ETI responses also encompass the dramatic “last-stand” defense called the hypersensitive response (HR), a form of localized programmed cell death, in which the plant strategically kills off regions that are infected by a pathogen in order to prevent spreading of the pathogen to other cells and tissues (Jones et al. 2024).

However, as the once clear boundaries between PTI and ETI continue to blur, and given the broad-spectrum activity of many of these compounds, it is increasingly evident that the compounds discussed in this review are tightly intertwined with systems that relate directly to both PTI and ETI responses, but also indirectly. One example is that during PTI activation, plants produce and secrete antimicrobial compounds into the surrounding environment to contain or eliminate the invading microbes. In a classic “effector” countermeasure, some pathogens attempt to disarm this response by targeting vesicle trafficking, thereby blocking the secretion of these defense compounds (Nomura et al. 2006; Xin and He 2013).

One important point we want to raise from this is that it is becoming evident that plant immunity is not dictated by a single signaling pathway or compound. Rather, it emerges from a complex mixture of overlapping signal activation, transduction, and integration that modulates suites—or modules—of compounds. While we are only beginning to understand this, it is clear that these processes are shaped by factors such as prior exposures, which may leave an epigenetic imprint, and by local and systemic signaling dynamics. One additional complication is that the plant's chemical response itself is best seen as flexible and multilayered—one that does not rely on a single silver bullet but instead on a diverse and context-dependent arsenal. It is still unknown how these sensing mechanisms converge with the production of distinct chemical cocktails, and much remains to be learned about their coordination and mode of action. Thus, the next sections, detailing various plant defense compounds, should be viewed not in isolation but considered as pigments on an artist's palette, where each class can give its own hue and texture necessary to counter a specific attack.

## The arsenal

Arabidopsis is part of the Brassicales plant order, containing numerous crops such as mustard, kale, radishes, and rapeseed. An intriguing aspect associated with this order is their highly diminished ability to form symbiotic associations with fungi, a characteristic that sets them apart from most other plant species (Anthony et al. 2020). One distinctive feature, which is likely associated with this lack of symbiotic connections, is their specialized sulfur (S) metabolism. This is integrated into the production of defense chemicals that contribute to the spicy mustard flavor often found in Brassica crops and that also interfere with symbiosis, for instance by inhibiting the formation of arbuscules in arbuscular mycorrhizal fungi (Trombley et al. 2025).

The underlying chemistry of this is a diverse array of amino acid-derived S-containing glucosides known as *glucosinolates* (GSLs). Over 132 different GSLs have been documented to occur across Brassicales (Agerbirk and Olsen 2012). Dependent on the amino acids from which they are derived, these GSLs can be classified into 3 main groups: aliphatic GSLs (AGSLs; derived from methionine, alanine, leucine, isoleucine, or valine), aromatic or benzenic GSLs (BGSLs; derived from tyrosine, and phenylalanine), and indolic GSLs (IGSLs, derived from tryptophan). GSLs are the most abundant defense compounds in Arabidopsis, and besides their culinary appreciation, much of our understanding of chemical defense systems comes from research focused on these compounds.

In addition to GSLs, Arabidopsis produces several other bioactive compounds derived from the same amino acids through parallel and often interconnected metabolic pathways. Among these, *camalexin*, *coumarins*, various *flavonoids* and *terpenoids* stand out for their documented antimicrobial roles. In the following sections, we summarize what is known about this arsenal in Arabidopsis, focusing specifically on those compounds that have been experimentally demonstrated to have antimicrobial activity. This, admittedly, biases the review toward the GSLs and similar compounds, but we look forward to research that gives deeper insight into these aspects of other classes of defense metabolites.

### Methionine-derived compounds

**Figure 1.**
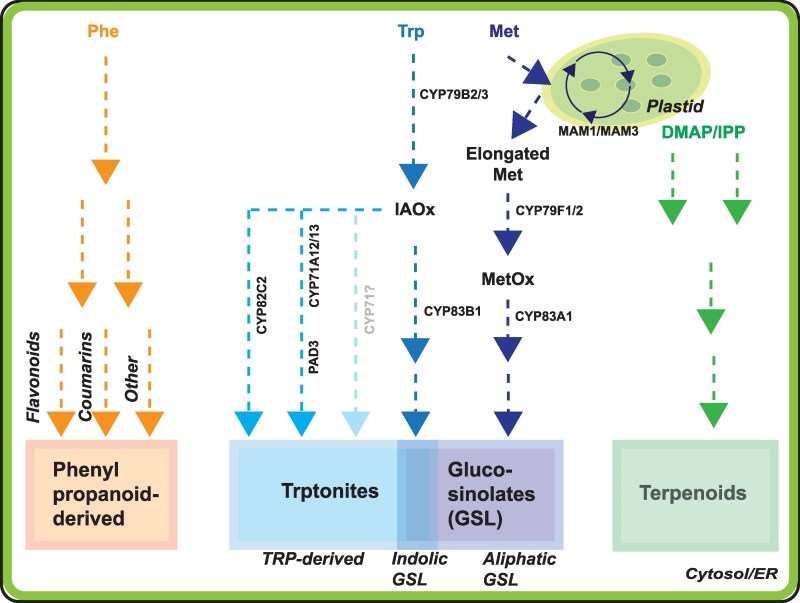
The interconnected biosynthetic pathways of Arabidopsis defense compounds. Most defense compounds are synthesized from the amino acid precursors phenylalanine (Phe), tryptophan (Trp) or methionine (Met). Phe is converted in a series of reactions into different compounds collectively termed phenylpropanoids. These form the basis for phenylpropanoid-derived compounds, which include flavonoids and coumarins, but also others, such as BGSLs and hydroxycinnamates. The compounds synthesized from Trp are collectively called trptonites, and their biosynthesis is initiated in an initial conversion step by CYP79B2 and CYP79B3. The most prominent trptonites are the IGSLs, which are displayed in the overlap but are indeed part of the group. IGSLs are produced in a series of reactions via the intermediate IAOx, which is the substrate for CYP83B1. IAOx serves as a branch point for trptonite biosynthesis, and the homologs CYP71A12 and CYP71A13 can funnel it toward camalexin (via PAD3) or hycanite (via CYP82C2) biosynthesis. Importantly, as there are several more CYP71 homologs in Arabidopsis, there may be many more trptonites (unknown compounds) that remain to be discovered. AGSLs are produced from Met, which is first chain elongated via MAM1/MAM3 in a plastid, such as the chloroplast. Similar to IAOx in the IGSL pathway, MetOx is a branch point for AGSL biosynthesis. CYP83A1 is the enzyme that funnels the substrate toward the GSL. In both cases, IGSLs and AGSLs, myrosinases perform the final activation step, to produce the active defense compound. Finally, terpenoids are synthesized from chloroplast-produced isoprene via DMAP/IPP through a series of reactions. Terpenoids include (E)-β-caryophyllene, α- and β-pinene, thalianin, arabidin, and DMNT.

Methionine forms the basis for several important metabolites, including the phytohormone ethylene and the vitamin biotin (Hacham et al. 2023). With regard to defense compounds in Arabidopsis (Col-0), methionine is the precursor for AGSLs (Fig. 1; Harun et al. 2020). One remarkable aspect of the pathway responsible for their production is the ability to utilize chain-elongated methionine to synthesize AGSLs consisting of up to 5 carbon atoms in the side-chain of short-chained AGSLs and in Arabidopsis, up to 8 in long-chained AGSLs (Textor et al. 2007; Fig. 1).

#### Aliphatic GSLs

Although we deliberately avoid focusing on herbivory in this review, it is important to mention that AGSLs were originally identified as chemoprotectants based on their differential toxicity toward generalist or specialist herbivores (Kjær 1960; Blau et al. 1978; Siemens and Mitchell-Olds 1996). This insight led to the identification of a transcription factor (TF) of the myoblastosis class (MYB), MYB28, which is transcriptionally induced by mechanical stimuli, mirroring the damage caused by feeding herbivores (Gigolashvili et al. 2007b; Sønderby et al. 2010). Ectopic *MYB28* expression enabled induction and overproduction of AGSL, which resulted in the understanding that AGSL accumulation correlates with protection against the lepidopteran herbivore *Spodoptera exigua* (Gigolashvili et al. 2007b). Evidence for microbial effects of AGSL was shown by preexposing roots to the nonpathogenic *Pseudomonas fluorescens* strain SS101, which induces systemic resistance responses, including the production of AGSL, IGSL, and coumarins, and enhanced resistance against the pathogenic bacterium *Pseudomonas syringae* pv *tomato* (Pst), as well as the insect *S. exigua* (van de Mortel et al. 2012). The AGSL 4-methylsulfinylbutyl GSL (4-MSB, also known as glucoraphanin) makes up over 60% of detected GLS in the Arabidopsis Col-0 accession (Unger et al. 2024). One key study demonstrated that an activated (see Box 1) 4MSB-derived product, 4-methylsulfinylbutyl isothiocyanate (4MSB-ITC; sulforaphane), accumulates in *Alternaria brassicicola*–infected leaves, exhibiting broad-spectrum antimicrobial activity against fungi and bacteria, including *Fusarium oxysporum*, *Neurospora crassa*, *Verticillium dahliae*, and *P. syringae* (Tierens et al. 2001). However, *glucosinolate mutant 1-1* (*gsm1-1*) plants, which harbor a mutation in the *METHYLTHIOALKYLMALATE SYNTHASE 1* (*MAM1*) gene, have a reduced ability to chain-elongate methionine and therefore predominantly accumulate 3-methylsulfinylpropyl. However, these mutant plants do not show an enhanced susceptibility to any of these potential pathogens in planta, except for *F. oxysporum* (Haughn et al. 1991; Kroymann et al. 2001; Tierens et al. 2001). At the same time, when the *gsm1* mutant is challenged with the insect herbivore *Spodoptera* littoralis, it is more susceptible to larval feeding and growth (Schlaeppi et al. 2008). Thus, while there is strong evidence that AGSLs, specifically 4MSB, act as insect repellents, their role for broad-spectrum antimicrobial activity is still unclear.

Box 1. Detonation systems: How plants safely deploy deadly weapons Loaded guns in homes are more likely to cause accidents than fend off intruders (Kellermann et al. 1998). Plants have evolved clever ways to store their weapons *safely* while remaining vigilant (Jones and Vogt 2001; Stathaki et al. 2024). Instead of keeping the defense compounds in their toxic form, plants can store weaponized substances as glycosylated, nonreactive versions. When trouble arises, beta-glucosidases (BGLUs) detonate the glucose-conjugated chemicals via rapid hydrolysis and convert them back into their dangerous form (Stathaki et al. 2024). One of the best-known examples of this “detonator system” is the so-called mustard-oil bomb in Arabidopsis. In damaged plant tissues, GSLs are quickly converted into reactive products, creating that famous spicy mustard flavor (Matile 1980). The BGLUs responsible for this are termed *myrosinases*, and come in 2 varieties: typical and atypical (Chhajed et al. 2020). Six myrosinases, THIOGLUCOSIDE GLUCOHYDROLASE (TGG) 1 to 6, have been identified (Barth and Jander 2006; Andersson et al. 2009). TGG1 and 2 are keys in activating GSLs in shoots, while TGG4-6 serve similar functions in root and flower defenses. *TGG3* is a pseudogene (Barth and Jander 2006; Andersson et al. 2009). Whereas the TGGs are important in Arabidopsis to fend off pests like spider mites and caterpillars (Barth and Jander 2006; Widemann et al. 2021), the atypical myrosinases are essential for broader immunity. Of these, BGLU26 (PENETRATION 2, PEN2) plays a crucial role in nonhost specific defenses. This atypical myrosinase breaks down indolic glucosinolates (IGSLs) to produce defense compounds that help defend against a large range of pathogens (Piślewska-Bednarek et al. 2018). BGLU23 (pyridine-3-carbinol 10-kDa protein, PYK10), another atypical myrosinase, has in vitro activity but currently no confirmed role in pathogen defense. It does, however, play a part in shaping the root microbiome and promotes beneficial microbial interactions (Sherameti et al. 2008; Basak et al. 2024). Other BGLUs, like BGLU21-23 and BGLU42, activate coumarins (e.g. converting scopolin to scopoletin), which also provides defense against pathogens like *Botrytis cinerea* and *Pseudomonas syringae* (Zamioudis et al. 2014; Stringlis et al. 2018). While this “detonator” system helps plants keep their deadly weapons safely stored and ready for action, there is still much to learn: Arabidopsis has at least 47 BGLUs, and many are likely responsible for activating other defense compounds. Future research should aim to match up these enzymes with their specific substrates and products.

Infection of Arabidopsis by the fungus *Sclerotinia sclerotiorum* triggers the AGSL-myrosinase system, which, interestingly, results in the transcriptional upregulation of key AGSL pathway genes and the rapid production of 4MSB-ITC (Stotz et al. 2011; Chen et al. 2020). Further, the *myb28*, double *myb28 myb29*, and double *tgg1 tgg2* mutants are all more susceptible to this fungus (Stotz et al. 2011; Chen et al. 2020). However, assessment of the antimicrobial properties of different AGSL-ITCs in vitro showed that the antifungal activity is dependent on the side-chain elongation, with long-chain aliphatic 8-methylsulfinyloctyl isothiocyanate (8MSOO-ITC), not 4MSB-ITC, showing the strongest activity against *Sc. sclerotiorum* (Stotz et al. 2011; Chen et al. 2020). Intriguingly, this insight also led to the discovery that *Sc. sclerotiorum* metabolizes 4MSB-ITC via an ITC hydrolase, encoded by a *sax* (*survival in Arabidopsis extracts*) gene, that converts ITC into products nontoxic to the fungus (Chen et al. 2020). Further, when the AGSL biosynthesis pathway is blocked by mutating nonessential residues in *CYP83A1*—an essential enzyme in AGSL formation—resistance against the powdery mildew fungus *Erysiphe cruciferarum* is enhanced (Weis et al. 2014). This is most likely due to the accumulation of the intermediate 5-methylthiopentanaldoxime, a substrate for CYP83A1, which is toxic to the fungus (Weis et al. 2014). In support of this, aldoximes, such as 5-MTPO, have been proposed as an alternative method by which the plant can provide fungal resistance (Lamberth 2024; Fig. 1). These studies thus demonstrate that it is not always the end-product of a certain GSL pathway that is the active substance, but that any intermediate can also have antimicrobial properties. Together with the fact that microbes have evolved ways to hydrolyze certain compounds, both observations are most likely a reflection of the molecular arms race between plants and microbes.

In the case of *Botrytis cinerea*, a role for AGSLs is less clear. Different studies have found no consistent antifungal activity of 4MSB-ITC on *B. cinerea* in vitro, and while infection of the plant induces AGSL production, mutants affected in their accumulation do not exhibit enhanced susceptibility (Tierens et al. 2001; Kliebenstein et al. 2005). However, the interaction of *B. cinerea* with Arabidopsis, as well as its effect on GSL metabolism, is highly dependent on the *B. cinerea* isolates used, as well as the Arabidopsis accessions tested (Kliebenstein et al. 2005, 2001). Remarkably, a recent study found that the most commonly studied *B. cinerea* isolate has an isolate-dependent detoxification mechanism that acts to export GSL breakdown products from the fungus (Vela-Corcía et al. 2019). So, again, the ongoing molecular arms race between plants and microbes has led to extreme specializations on both sides, highlighting the need for us, as scientists, to be exceedingly specific when describing and interpreting results and observations.

Another interesting example, aimed at elucidating a molecular mechanism of AGSLs, comes from the case of the bacterium *P. syringae* pv *tomato DC3000*, which is sensitive to both allyl- and 4MSB-ITC in vitro, but not in vivo (Tierens et al. 2001; Fan et al. 2011; Wang et al. 2020). Although the authors did not test the allyl-ITC sensitivity in vivo (since it is not produced by Col-0), they found that 4MSB-ITC inhibits the bacterial Type III secretion system (TTSS) by directly modifying the TTSS co-factor HrpS, which reduces the pathogenicity of the bacterium (Wang et al. 2020). So while this work does not rule out a differential effect of allyl-ITCs, it is in line with their results that pathovars not adapted to Arabidopsis are more sensitive to 4MSB-ITC, while virulent pathovars, such as *tomato DC3000* and *maculicola* (*Psm*) ES4326 can grow in the presence of 4MSB-ITC (Fan et al. 2011). Fascinatingly, this is due to the presence of several *sax* genes in the virulent strains, including a bacterial homolog of the *Sc. sclerotiorum sax* gene, which can detoxify 4MSB-ITC (Fan et al. 2011; Chen et al. 2020). Indeed, Col-0 mutants devoid of AGSLs, such as the *myb28 myb29* double mutant, display an enhanced susceptibility to infection by the pv *tomato DC3000*, which can be rescued by the application of 4MSB-ITC (Wang et al. 2020). A recent study characterized one of the *sax* genes as encoding an efflux pump that specifically contributes to Arabidopsis shoot colonization (Russ et al. 2024). This efflux pump is prevalent across the *Pseudomonadota* pangenome, yet benefits the bacterial association with only a small subset of Arabidopsis accessions (Russ et al. 2024). We are excitedly waiting for future in vivo studies revealing how more species *sax* genes affect survival on plants containing different GSL classes.

In summary, as part of the arms race between Arabidopsis and *P. syringae*, certain bacterial strains have gained the ability to detoxify 4MSB-ITC, while the plant, in turn, can inhibit the bacterial TTSS, reducing pathogenicity and countering bacterial adaptation (Wang et al. 2020). This appears to be particularly important when it comes to the colonization of leaves by bacteria to form a functional microbiota, as the diversity of AGSLs plays an important role in shaping the distinct community found in shoots (Unger et al. 2024). Combined, these examples demonstrate a clear effect of different AGSLs as defense compounds (Table 1). However, they also highlight that the contribution of individual AGSLs to overall plant resistance is not easy to assess due to their biosynthetic interconnectedness as well as genetic variation in both plants and their enemies.

**Table 1.** Microbes and their identified effective AGSL weapons in Arabidopsis

Species	Type	Sensitive toward	Relevant mutants
*Pseudomonas syringae* pv *tomato*	Bacterium	AGSLs (4MSB)	*myb28 myb29*
*Erysiphe cruciferarum*	Fungus	5-MTP	*cyp83a1*
*Fusarium oxysporum*	Fungus	AGSLs	*gsm1-1*
*Sclerotinia sclerotiorum*	Fungus	AGSLs (8MSO-ITC, 4MSB-ITC)	*myb28*; *myb28 myb29; tgg1 tgg2*
*Botrytis cinerea*	Fungus	AGSLs (ITCs)	

As you might surmise from the above, we could continue discussing AGSLs, as these compounds are remarkable. However, we will conclude this section by emphasizing our main point: it is clear that this class of compounds plays diverse roles in the Arabidopsis defense system by acting against insect herbivores *and* microbial pathogens. Their regulatory pathways, breakdown products, and dynamic interactions with pests and pathogens demonstrate their importance in Arabidopsis defense strategies and the ongoing evolutionary arms race. Thus, AGSLs serve as a good example of how creative and versatile plants are in defending themselves at multiple levels.

### Tryptophan-derived compounds (trptonites)

Tryptophan (Trp) is an essential amino acid in plants, positioned at the intersection of developmental- and defense-related metabolism. It is the precursor molecule for the phytohormone auxin, as well as several defense compounds, which we collectively refer to here as trptonites (Fig. 1). These include IGSLs, the cyanogenic glycoside 4-OH-ICN (4-**hy**droxyindole-3-**ca**rbonyl **nit**ril**e**; *hycanite*), and the 3-thiazol-2-yl-indole known as *camalexin* (McDanell et al. 1988; Hansen and Halkier 2005). Trptonites are especially well-known in Arabidopsis, as several key nodes in the biosynthetic network (Fig. 1) can be utilized to genetically knock out all of these compounds at once, specific sub-groups, or individual compounds to assess their function in plant immunity.

#### Indolic GSLs

Similarly to the AGSLs, IGSLs are regulated by MYB-class TFs. MYB51 was identified as a key transcriptional activator of IGSL biosynthesis (Gigolashvili et al. 2007a). Again, herbivores will have to be mentioned, since overexpression of *MYB51* elevates IGSL levels and enhances resistance to the lepidopteran herbivore *S. exigua* (Gigolashvili et al. 2007a). Thus, like AGSLs, IGSLs were primarily assumed to function in herbivory defense, and the *cyp79B2 cyp79B3* double mutant, devoid of all trptonites, is indeed more susceptible to insect herbivory (Schlaeppi et al. 2008; Müller et al. 2010). Further work on the role of IGSLs in herbivory defense has established a link between jasmonic acid biosynthesis and IGSL production (Zhurov et al. 2014; Widemann et al. 2021; Stahl et al. 2022). Notably, in the case of *Tetranychus urticae*, activation of a modified IGSL, 4-methoxy-3-indolylmethyl GSL (4MOI3M), relies on the myrosinases TGG1 and TGG2, and not PEN2, which may suggest functional overlap between myrosinases (Widemann et al. 2021). Importantly, in Arabidopsis–microbe interactions, IGSLs are essential as both antimicrobials and signaling components. Treatment of Arabidopsis seedlings with exogenous flg22 peptide results in the activation of PTI, including the deposition of callose in the cell wall. Clay et al. (2009) showed that flg22 furthermore triggers expression of *MYB51* and downstream IGSL biosynthesis. This activation of IGSL biosynthesis via flg22 leads to the formation of 4MOI3M. Interestingly, inhibiting 4MOI3M production in *pen2* mutants not only results in IGSL depletion but also inhibits the other basal immune responses linked to flg22, including callose deposition (Clay et al. 2009). Thus, IGSLs are integral as antimicrobial outputs of the immune response system but remarkably also work as regulators within the signaling pathway launching the full basal immune response (Clay et al. 2009).

Beyond their role in flg22-induced basal immunity, 4MOI3M-derived breakdown products are also involved in direct defense against fungal pathogens, including *Blumeria graminis* and *Erysiphe pisi* (Lipka et al. 2005; Bednarek et al. 2009). Plants with nonfunctional versions of the myrosinase *pen2* are more readily penetrated by hyphae from both these fungi (Lipka et al. 2005). In response to *B. graminis* infection, raphanusamic acid (RA) and indol-3-ylmethylamine (I3A) are produced at high levels—an induction lost in *pen2* mutants, which suggests that these 2 compounds are the toxic, activated forms of the IGSLs released by the PEN2 myrosinase (Bednarek et al. 2009). Conversely, only 4MOI3M accumulates in *pen2* mutants, supporting that it is a substrate for PEN2, in agreement with the findings from Clay et al. (2009). Thus, IGSL-derived products such as RA and I3A restrict fungal pathogen entry into the plant (Bednarek et al. 2009).

IGSLs also play a key role in protection against the vascular wilt fungus *Verticillium longisporum* (Fröschel et al. 2019). Overexpression of the genes encoding the ERF96 and ERF102 to ERF107 TFs enhances resistance to *V. longisporum*. Interestingly, single *erf* mutants do not show enhanced susceptibility, indicating the utility of this gain-of-function approach. Enhanced resistance in these overexpressing lines depends on the transcriptional upregulation of several IGSL-associated genes, including *MYB51* and *CYP81F2*, as well as the camalexin biosynthesis gene *PAD3* (Fröschel et al. 2019). Consistent with this, *cyp81f2* mutants are more susceptible to colonization by *V. longisporum*, though not as severely as the *cyp79b2 cyp79b3* double mutant, pointing to a role for other trptonites (Fröschel et al. 2019). Indeed, co-contribution of different metabolites from shared enzymatic machinery to the same defense reaction is 1 convoluting factor when investigating trptonite-based resistance. A prominent example of this comes from studying the resistance against the oomycete *Phytophthora brassicae* (Schlaeppi et al. 2010). While removing the ability to produce IGSLs or camalexin alone does not lead to susceptibility, the loss of all trptonites in the *cyp79b2 cyp79b3* double mutant—or the simultaneous elimination of camalexin and activated IGSLs like RA and I3A in the *pad3 pen2* double mutant—significantly increases penetration and infection (Schlaeppi et al. 2010). Thus, in many cases, complex mixtures of trptonites likely work synergistically to provide immunity.

#### Camalexin

The defense compound camalexin shares biosynthetic steps with IGSLs (Fig. 1). Consistent with this overlap, the *cyp79B2 cyp79B3* double mutant cannot produce camalexin (Hull et al. 2000). Camalexin was first identified in *Camelina sativa* when infected by *Alternaria brassicae* and in Arabidopsis when challenged with *P. syringae* pv *syringae* (Browne et al. 1991; Tsuji et al. 1992). In the latter case, an HR and local camalexin accumulation were found to be responsible for restricted bacterial growth (Tsuji et al. 1992). Despite serving as a hallmark of pathogen-induced responses, the exact role of camalexin in defense is still a subject of debate, particularly regarding its interactions with different pathovars of *P. syringae* and *B. cinerea* (Glazebrook and Ausubel 1994; Thomma et al. 1999b; Zhou et al. 1999; Ferrari et al. 2003; Denby et al. 2004; Rowe and Kliebenstein 2008). Several factors complicated this discussion: For one, in the case of pathogens such as *B. cinerea* and *Sc. sclerotiorum,* different pathovars have detoxification mechanisms against certain compounds, including camalexin, which can result in contrasting results when different isolates are used (Stefanato et al. 2009; Pedras et al. 2011; Chen et al. 2020). Secondly, as mentioned above, the interconnectedness of camalexin biosynthesis with the other trptonites—in particular currently uncharacterized compounds that are difficult to detect but share common synthesis machinery—likely causes confusion when assigning a role to camalexin.

Mutant studies have provided key insights into camalexin biosynthesis and its defense roles. The *PHYTOALEXIN DEFICIENT 1-4* (*PAD1-4*) mutants, identified in a screen aimed at identifying disease-susceptible plants (Glazebrook and Ausubel 1994), have been particularly useful. While all 4 described *pad* mutants show reduced camalexin levels, only *PAD3* turned out to be a *bona fide* camalexin biosynthesis gene (Zhou et al. 1999; Schuhegger et al. 2006). *PAD3* encodes CYP71B15, the enzyme that converts dihydrocamalexic acid to camalexin (Schuhegger et al. 2006). PAD1 and PAD2, in contrast, are linked to proteasomal ubiquitination and proteolysis, respectively, while PAD4 is involved in salicylic acid signaling (Monsalvo et al. 2024). The involvement of PAD3 in defense is especially evident against *B. cinerea*, as the *pad3* mutant exhibits heightened susceptibility (Ferrari et al. 2003, 2007). WRKY33, a TF central to camalexin synthesis, directly drives the expression of *PAD3* and *CYP71A12* in response to infection by *B. cinerea* (Mao et al. 2011; Birkenbihl et al. 2012, 2017). WRKY33 itself is activated via cooperative phosphorylation by the MPK3/6 and CPK5/6 pathways, which enhances its DNA-binding and activation activity, respectively (Zhou et al. 2020). Additionally, WRKY33 integrates jasmonic acid and ethylene signals via the ethylene response factors 1 and 27 (ERF1/27), which directly activate *WRKY33* expression and thereby camalexin synthesis (Thomma et al. 1998, 1999a; Li et al. 2022; Zhou et al. 2022). The importance of jasmonic acid for camalexin induction was further demonstrated in a study assessing camalexin production and disease severity in jasmonic acid biosynthesis and signaling mutants, and in response to infection with different *B. cinerea* isolates (Rowe et al. 2010). Plants depleted of jasmonic acid generally do not accumulate camalexin and show enhanced disease severity (Rowe et al. 2010). However, intriguingly, some *B. cinerea* isolates are still able to induce camalexin production, even in the absence of any jasmonic acid biosynthesis and signaling, albeit at reduced levels, indicating a so far unidentified regulatory mechanism (Rowe et al. 2010). Camalexin synthesis is strongly linked to inducible signaling and is considered a robust output from both PTI and ETI (Nguyen et al. 2022) and it is possible that the unknown mechanism(s) link up to systems that underlie this connection.

Although camalexin production was originally identified as a response to bacterial elicitation, it is now primarily associated with fungal and oomycete resistance. Examples of this include *A. brassicicola*, *Ph. brassicae*, *B. cinerea*, *Leptosphaeria maculans*, or *Sc. sclerotiorum* (Thomma et al. 1999b; Ferrari et al. 2003; Bohman et al. 2004; Schlaeppi et al. 2010; Stotz et al. 2011). The standard method to investigate susceptibility is to score disease symptoms after manual infection of wild-type and *pad3* mutant plants in artificial laboratory conditions. A more complex biotic setting for infection assays may offer further valuable insights into camalexin function, particularly its influence on bacterial communities in the rhizosphere.

An example of this comes from using sulfatase activity as a proxy for microbial activity in the soil. Through a genome-wide association study (GWAS), a study involving 172 Arabidopsis accessions identified *CYP71A27* as responsible for changes in microbial soil activity (Koprivova et al. 2019). *CYP71A27* is associated with camalexin synthesis and is specifically expressed in the root, suggesting that camalexin production may also contribute to belowground protection. Notably, *CYP71A27* expression is suppressed by the beneficial *Pseudomonas* sp. CH267 but upregulated by the pathogenic *Burkholderia glumae* PG1 (Koprivova et al. 2023). Surprisingly, CYP79A27 may be metabolically inactive, and instead, it plays a role in regulating camalexin-related signaling (Koprivova et al. 2025). Nonetheless, *cyp71A27* mutant plants (or natural accessions with reduced CYP71A27 activity) produce less camalexin, leading to altered rhizosphere dynamics and loss of growth-promoting effects of beneficial bacterial strains such as *Pseudomonas* sp. CH267 (Koprivova et al. 2019). Importantly, it remains unclear whether camalexin directly inhibits *Bu. glumae* PG1 or if this is influenced by indirect effects, such as reshaping the rhizosphere microbiome. Testing the susceptibility of camalexin-deficient mutants to *Bu. glumae* PG1 in natural conditions, and perhaps in combination with different accessions, will help clarify whether camalexin acts directly against pathogens or indirectly through ecological interactions (Koprivova et al. 2019, 2023, 2025). Moreover, another intriguing interpretation of this complex interaction between camalexin and rhizosphere-dwelling microorganisms is that community-wide emerging properties affect camalexin production. In fact, this was recently demonstrated to occur via volatiles in situations where a 16-member synthetic community was grown in direct or indirect contact with Arabidopsis plants (Türksoy et al. 2025).

Camalexin has also been implicated in the defense against the protist Phytomyxea root-parasite *Plasmodiophora brassicae* (Lemarié et al. 2015). The natural accession Bur-0 (Burren) shows a strong induction of camalexin biosynthesis gene expression in response to infection, which results in high levels of camalexin in the root and a reduced growth of the parasite. Similarly, the *pad3* mutant in the Col-0 background is also more susceptible to *Pl. brassicae* than the wild type, further implicating camalexin as responsible for suppressing *Pl. brassicae* growth (Lemarié et al. 2015).

The elegant studies highlighted above show that camalexin is more than just a defense compound targeting specific pathogens; it plays a broader role in shaping plant–environmental interactions, influencing the entire plant microbiome (Table 2). Understanding the role of camalexin and the trptonites within natural settings that preserve microbiomes and account for organ-specific dynamics will provide a more comprehensive and accurate depiction of their ecological roles. In fact, this is true for any of the described chemical weapons in this review.

**Table 2.** Microbes and their identified effective trptonite weapons in Arabidopsis

Species	Type	Sensitive toward	Relevant mutants
*Pseudomonas syringae* pv *tomato*	Bacterium	IGSLs; hycanite; camalexin	*cyp79b2 cyp79b3*; *cyp71a12*; *cyp71a13; cyb81f2; pen2; myb51; cyp82c2*; *pad3*
*Pseudomonas syringae* pv *syringae*	Bacterium	Camalexin	
*Botrytis cinerea*	Fungus	Hycanite; camalexin	*cyp79b2 cyp79b3*; *cyp71a13*; *cyp82c2*; *pad3*
*Blumeria graminis*	Fungus	IGSLs (RA, I3A)	*pen1-1; pen2-1; pen2-2; pen1-1 pen2-1*
*Erysiphe pisi*	Fungus	IGSLs (4MOI3M)	*pen1-1; pen2-1; pen1-1 pen2-1*
*Verticillium longisporum*	Fungus	IGSLs; camalexin	*cyp79b2 cyp79b3*; *cyp81f2; pad3*; *erf102 erf103*
*Colletotrichum gloeosporioides*	Fungus	IGSLs	*pen2*
*Plectosphaerella cucumerina*	Fungus	IGSLs	*pen2*
*Alternaria brassicicola*	Fungus	Hycanite; camalexin	*cyp79b2 cyp79b3*; *cyp71a13*; *cyp82c2*; *pad3*
*Leptosphaeria maculans*	Fungus	Camalexin	*pad3; esa1; acd1-20*
*Sclerotinia sclerotiorum*	Fungus	Camalexin	*cyp79b2 cyp79b3*; *pad3*
*Phytophthora brassicae*	Oomycete	IGSLs (I3A) ; camalexin	*cyp79b2 cyp79b3; pen2* ; *pad3; myb51*
*Plasmodiophora brassicae*	Phytomyxea	Camalexin	*pad3;* Bur-0 accession

#### Hycanite (4-OH-ICN)

Arabidopsis synthesizes another trptonite termed hycanite in response to infection by *P. syringae* pv. *tomato* DC3000, *B. cinerea*, and *A. brassicicola* (Liu et al. 2010; Rajniak et al. 2015). The key biosynthesis gene *CYP82C2* was identified through a remarkable study. In this work, the authors used microarray-based analyses to identify expression of *CYP82C2* as strongly upregulated under a wide variety of biotic stress conditions. By comparing accumulating metabolites of wild-type (Col-o) and *cyp82c2* mutant plants following infection with *P. syringae* pv. *tomato* DC3000, 11 compounds were found to be differentially produced between the genotypes. This metabolic blockage enabled the reconstruction of the complete biosynthetic pathway of hycanite (Rajniak et al. 2015). Further mutant analysis showed that plants lacking *cyp71A12* and *cyp82C2* were more susceptible to infection by *P. syringae* pv. *tomato* DC3000, and *A. brassicicola*, which could be complemented by treatment with exogenous hycanite (Rajniak et al. 2015). The authors further showed that plants carrying mutations in *cyp71A12* and *cyp82C2* are also more susceptible to infection by *B. cinerea*, but this effect could not be replicated in a later study, where the lesion sizes of the 2 mutants were comparable to the Col-0 wild type (Celine Caseys et al. 2024). Thus, there may be some unknown conditionalities to the effect of hycanite on *B. cinerea*.

Hycanite and camalexin share the earliest step of trptonite biosynthesis, the conversion of Trp into IAOx (Fig. 1). However, it is less clear what happens in the following. Initially, it was assumed that the conversion of IAOx into indole cyanohydrin, catalyzed redundantly by CYP71A12 and CYP71A13, was still shared between both compounds, and that their pathways only diverge at this point (Rajniak et al. 2015). However, more recently, it was suggested that the 2 CYP71 homologs may form the branch point, with CYP71A13 predominantly feeding into the camalexin biosynthesis pathways, while CYP71A12 may reroute the pathway toward the synthesis of 4OH-ICN (Barco et al. 2019; Pastorczyk et al. 2020). The presence of numerous CYP71 paralogues in Arabidopsis therefore raises the intriguing possibility that each of them can funnel the biosynthetic pathway toward a specific output, and that additional trptonites therefore also remain undiscovered (Fig. 1). However, until this potential role for CYP71 proteins has been studied in detail *in planta*, this question cannot be definitively resolved. We very much look forward to reading the outcome of future studies that dig into these aspects.

### Phenylalanine-derived compounds (phenylpropanoids)

Phenylpropanoid metabolism is responsible for the biosynthesis of a diverse array of metabolites derived primarily from the amino acid phenylalanine (Fig. 1). The pathway begins with the deamination of phenylalanine by the phenylalanine ammonia lyases, thereby yielding trans-cinnamic acid. This step represents the entry point into the phenylpropanoid pathway and is tightly regulated in response to developmental cues and environmental stimuli. Trans-cinnamic acid undergoes hydroxylation and methylation reactions to produce intermediates like *p*-coumaric acid, which serves as a precursor for a variety of phenylpropanoids, including coumarins, flavonoids, anthocyanins, and others. These metabolites act in several plant pathways, including those involved in plant defenses, but perhaps most importantly, the phenylpropanoid pathway also gives rise to hydroxycinnamic acids and lignin, which forms the basis for wood formation.

#### Benzenic GSLs

From here on, we promise not to mention (much) more about GSL and keep (most of) our focus on other compounds. However, we must mention that an interesting crossover occurs in Arabidopsis that links the GSL pathway with phenylpropanoid metabolism. Two classes of BGSLs can be detected in Arabidopsis. One is directly synthesized from phenylalanine while the other is synthesized by conjugation of benzenoids or phenylpropanoids to a hydroxy or sugar moiety on AGSLs (Lee et al. 2012; Zhang et al. 2015). These both appear to be seed- and seedling-specific GSLs (Kliebenstein et al. 2007). So far, no BGSL antimicrobial activity has been reported in Arabidopsis, but they may provide protection upon seedling germination.

#### Coumarins

When it comes to the “core” phenylpropanoid metabolism, one of the best-studied examples in connection to the antimicrobial angle of this review is the coumarins. Coumarin synthesis is mainly initiated by the key enzyme FERULOYL-CoA 6′-HYDROXYLASE1 (F6′H1) (Schmid et al. 2014). *F6′H1* is positively regulated by the pathogen-responsive MYB TFs MYB15 and MYB72 (Schenke et al. 2011; Stringlis et al. 2018). Although the role of coumarins in shoots remains unclear, they are well-studied as plant-derived siderophores—compounds secreted into the soil by plant roots to mobilize otherwise unavailable iron for uptake by chelation or reduction (Vélez-Bermúdez and Schmidt 2023; Paffrath et al. 2024). Upon elicitation, coumarins with antimicrobial function are secreted from the root into the rhizosphere (Stringlis et al. 2019). This may serve to shape the root microbiome by enriching it with plant-beneficial soil-dwelling bacteria, which, particularly under iron-limiting conditions, can improve iron uptake (Voges et al. 2019; Harbort et al. 2020 ). Interestingly, some of the plant-beneficial bacteria that respond to the presence of coumarins produce their own redox-active metabolites called phenazines. Like coumarins, phenazines facilitate iron reduction, further enhancing its availability to the plant (McRose et al. 2023). Thus, the antimicrobial activity of coumarin could, at least in part, also be an indirect effect of its microbiome-altering role (Table 3). It is important to note, however, that we are only beginning to understand the complex underground world of plants, where soil characteristics, microbes, and nutrient availability influence and connect to each other. Nonetheless, we expect this to be an important area of research, particularly for its unique position at the interface between root development, immunity, and ecophysiology.

**Table 3.** Microbes and their identified effective coumarin weapons in Arabidopsis

Species	Type	Sensitive toward	Relevant mutants
*Fusarium oxysporum*	Fungus	Coumarin (Scopoletin)	*bglu42*; *myb72*
*Verticillium dahliae*	Fungus	Coumarin (Scopoletin)	*bglu42*; *myb72*
*Verticillium longisporum*	Fungus	Sinapoyl esters	*fah1-2*
*Pseudomonas syringae* pv *tomato*	Bacterium	Coumarin (Scopoletin)	*f6′h1*; *myb15*; *myb72*; *bglu42*

The primary coumarins secreted by Arabidopsis include esculetin, scopoletin, fraxetin, and sideretin, with scopoletin receiving the most attention for its defense-related roles (Stringlis et al. 2019). In healthy plants, scopolin and scopoletin are present in the root at moderate levels. It is thought that coumarins are stored in vacuoles in the form of the glucosylated scopolin, which is then rapidly converted into scopoletin and secreted upon pathogen attack (see Box 2) (Bednarek et al. 2005; Stringlis et al. 2019; Robe et al. 2021). This conversion is mediated by the β-glucosidases BGLU21, BGLU22, BGLU23, and BGLU42 (see Box 3; Ahn et al. 2010; Stringlis et al. 2018). For example, infection by *Globisporangium sylvaticum* (formerly *Pythium sylvaticum*) reduces scopolin levels, likely due to scopoletin formation, though this increase has not been shown in the same study (Bednarek et al. 2005). However, elicitation of the plant with flg22 did result in increased scopoletin levels in the root, as well as secretion thereof, indicating that this elicitor-driven conversion of scopolin to scopoletin does indeed happen (Schenke et al. 2011).

Box 2. How to put your weapons where they are needed Plants do not randomly make and detonate chemical weapons—they are also masters of logistics, actively transporting their defensive compounds to where they are most needed (Andersen et al. 2013; Hunziker et al. 2021). Arabidopsis and its GSLs take the spotlight again with glucosinolate (GSL) transporters (GTRs), particularly GTR1 and GTR2 (Andersen et al. 2013; Hunziker et al. 2021). These members of the NRT/PTR transporter family manage the movement of aliphatic GSLs (AGSLs) between source and sink tissues (Nour-Eldin et al. 2012; Andersen and Halkier 2014). AGSLs are synthesized in rosette leaves and roots. GTR1/2 orchestrate AGSL loading into the vasculature in leaves, and prevent excessive loss from roots, ensuring these compounds are retained there. From these source and storage sites, the AGSLs are transported to especially vulnerable tissues, such as young leaves, to provide additional protection (Brown et al. 2003; Hunziker et al. 2021). Accordingly, *Spodoptera littoralis*, a herbivore adapted to Arabidopsis, is more likely to feed on old leaves, while avoiding young leaves—a pattern lost in the *gtr1 gtr2* double mutant (Hunziker et al. 2021). While it should not be excluded that the transporters also modulate systemic immune signaling, such a role has, to our knowledge, only been demonstrated for aglycone breakdown products of GSLs that can depolarize membranes and induce inter-organ electrical priming (Gao et al. 2023). GSL transporters are not the only logistical workhorses. Other compounds, like the tryptophan-derived camalexin, rely on pleiotropic drug resistance (PDR) transporters such as PDR6, PDR8 (PEN3), and PDR12 for secretion during attacks (Han et al. 2024). Similarly, coumarins like scopoletin are secreted by the transporters PDR8 and PDR9, and root-derived scopoletin can reach the shoot of the plant (Robe et al. 2021; Stassen et al. 2021). This indicates that coumarins can be loaded into the phloem by transporters, similar to the GTRs (Robe et al. 2021). However, so far, research has been limited to the identification of a few transporters, particularly those associated with GSL transport. To fully understand the mechanisms underlying principles controlling the distribution of defenses, it will be important to also focus on other compounds in the future and how this is coordinated with GSL transport.

Box 3. How do plants store weapons strategically? Plants, often underestimated as static and guileless, are tactical geniuses in chemical warfare. Living in resource-scarce, ever-changing environments, plants must strategize their deployment of resources (Neilson et al. 2013). One way to achieve this is to anticipate attacks and prepare minefields that hinder enemy progress accordingly. Gymnosperms produce terpene- and alkaloid-rich resins distributed via a duct system for targeted deployment (Trapp and Croteau 2001). In Arabidopsis, S and M cells are adjacent to the phloem in stems, a typical position for sap-sucking pests, and thus serve as strategic deposition/detonation sites (Koroleva et al. 2000; Andréasson et al. 2001; Hunziker et al. 2019). S cells are named for their high sulfur (S) concentration, which is due to the GSLs stored in them (Koroleva et al. 2000). M cells (myrosin cells) contain high myrosinase concentrations (Xue et al. 1995; Andréasson et al. 2001). The S and M cells around the phloem are specialized defenses protecting the plant vasculature. If a pathogen enters the vasculature, the plant is in trouble as this not only allows the microbe to consume sugars, it also provides free access throughout the entire plant. Storing these 2 reactive compounds in separate, specialized cells, makes this an ideal example for the detonator systems to protect the vasculature (Box 1). Chewing of herbivores on the stem tissue leads to tissue disruptions and release of the GSLs and myrosinases from the S and M cells, which immediately react to produce active GSL forms. M cells were initially described in 1890, and by 1913, researchers had connected their co-location with S cells to plant defense (Guignard 1890; Peche 1913). Tissue damage in these areas correlated with the strong mustard taste that deters herbivores. Tissues devoid of M cells still have myrosinase activity (Iversen et al. 1979; Matile 1980). Indeed, tissue separation is not the only mechanism for the spatial separation of GSL and myrosinase: In other cell types, GSLs are stored inside the vacuole, while myrosinases are tethered to structures in the cytosol (Matile 1980; Matsushima et al. 2003; Lipka et al. 2005; Fuchs et al. 2016). As pathogen-induced cell damage likely also damages the tonoplast, this results in the release of GLSs into the cytosol, where they are immediately hydrolyzed by the myrosinases. The distributions of these systems related to adaptive resistance require further investigation, especially in roots, where little is known about the myrosinase system.

Scopoletin can provide protection against both bacterial and fungal pathogens. The *myb15* mutant displays increased susceptibility to *P. syringae* pv *tomato* (Schenke et al. 2011; Chezem et al. 2017). Similarly, colonization of the plant by the nonpathogenic *P. fluorescens* strain SS101 induces scopoletin production, thereby improving resistance to *P. syringae* pv *tomato* (van de Mortel et al. 2012). Other beneficial microbes can also modulate scopoletin production and systemic resistance. One example is colonization of roots by *Pseudomonas simiae* WCS417, which induces *MYB72* expression and activates expression of *F6′H1* and *BGLU42*, thereby leading to scopoletin production (Zamioudis et al. 2014; Stringlis et al. 2018). Interestingly, this process enhances both iron acquisition and systemic resistance (Zamioudis et al. 2014). Plants overexpressing BGLU42 show increased resistance toward infection by *B. cinerea*, *P. syringae* pv. *tomato* DC3000, and *Hyaloperonospora arabidopsidis* while the *bglu42* mutant is more sensitive to infection by *P. syringae* pv. *tomato* DC3000. It remains to be tested if this affects the turnover of other compounds such as the GSLs and how this may influence the observed responses.

Scopoletin also exhibits antifungal activity in vitro, as it inhibits the growth of *F. oxysporum* and *V. dahliae* in plate assays (Stringlis et al. 2018). Interestingly, some plant-beneficial fungal endophytes have developed the ability to convert scopoletin into the coumarin esculetin, thereby evading the antifungal effects (Van Dijck et al. 2025). Growth of *Macrophomina phaseolina* is initially also inhibited by the plant-secreted scopoletin, but as the fungus converts this into esculetin, it gradually returns to normal growth (Van Dijck et al. 2025). Further, in a twist of interconnectedness, the fungus-produced esculetin then acts as an iron-mobilizing catechol in the soil, thereby supporting the plant in iron acquisition and enabling it to grow under iron-limiting conditions (Van Dijck et al. 2025).

As exemplified by this recent study, the disentanglement of how coumarins act in both iron acquisition and antimicrobial defense is challenging. It is plausible that the different coumarins have distinct roles, with some acting primarily as siderophores and others as antimicrobial agents. Alternatively, rather than individual coumarins serving separate functions, they may collectively work to shape the plant's soil microbiome. We would not be surprised if this cooperative action could involve attracting microbes that enhance iron bioavailability, while simultaneously deterring pathogens, thereby actively promoting the dominance of beneficial microbes. Moreover, elucidating how these effects interplay with other compounds such as GSLs or trptonites may reveal novel mechanisms used in situ for microbial manipulation.

#### Hydroxycinnamates

Metabolic fingerprinting has shown that several phenylpropanoids, including sinapoyl esters and coniferin, are produced in response to plant infection by the fungus *V. longisporum* (König et al. 2014). Sinapoyl esters are part of the hydroxycinnamates, which are produced from phenylalanine via p-coumarate. A key enzyme in their biosynthesis is FERULATE-5-HYDROXYLASE (F5H), which, confusingly, is the disrupted gene in the *fah1* mutant (Chapple et al. 1992). This mutant is more susceptible to infection by *V. longisporum*, further supporting that hydroxycinnamates have antifungal properties (König et al. 2014). This study further found that overexpression of UGT72E resulted in increased coniferin production, and these plants were more resistant to *V. longisporum* (König et al. 2014). Thus, it appears that hydroxycinnamates and coniferin play overlapping roles in defense against *V. longisporum* (König et al. 2014). However, we still have a lot to learn when it comes to hydroxycinnamates as potential defense compounds.

#### Flavonoids

Naringenin**—**Naringenin displays antifungal activity, as demonstrated by its ability to inhibit the growth of fungi such as *Plectosphaerella cucumerina*, *F. oxysporum* and *Colletotrichum higginsianum* in vitro (Camargo-Ramírez et al. 2018). Additionally, pretreating plants with naringenin activates multiple defense pathways, including salicylic acid signaling and MPK3/6 activation, which enhances resistance to *P. syringae* pv. *tomato* DC3000 infection (An et al. 2021). While a direct role in plant defense remains to be described, naringenin may also serve as a signaling molecule that induces or primes defense mechanisms. However, a more thorough functional analysis of the role of these compounds, including mutants in flavonoid biosynthesis, is needed before any role in plant defense can be properly assigned.

Kaempferol**—**Camargo-Ramírez et al. (2018) investigated the role of the microRNA miR858 in regulating plant susceptibility to colonization by *Plectosphaerella cucumerina*, *F. oxysporum*, and *Colletotrichum higginsianum*. Their work identified that overexpressing miR858 increases susceptibility, while silencing this transcript has the opposite effect (Camargo-Ramírez et al. 2018). These changes were further found to be linked to activation (silenced miR858) or repression (overexpressed miR858) of biosynthetic genes and corresponding accumulation or depletion of flavonoids. Furthermore, kaempferol was shown to inhibit fungal growth in vitro (Camargo-Ramírez et al. 2018). While these findings suggest a connection between these defense compounds and fungal colonization, mechanistic evidence for this is still lacking.

One additional point is that the variety of flavonoids is increased to dizzying amounts of different compounds, since kaempferol is further converted to quercetin and isorhamnetin which together with anthocyanidins, proanthocyanidins, and neolignans can be conjugated to sugars, malate, and/or hydroxycinnamates (Yonekura-Sakakibara et al. 2019). We look forward to reading what for sure will be exciting studies from researchers brave enough to embark on the world of flavonoid-microbe associations.

### Terpenoids

Terpenoids are derived from subunits of isoprene, which is synthesized in the chloroplasts via the methyl-erythritol 4-phosphate/nonmevalonate pathway. One of the 2 end-products, dimethylallyl pyrophosphate, is cleaved by the enzyme isoprene synthase to produce isoprene and diphosphate. Isoprene forms the basic building block for terpenes and terpenoids (oxygenated terpenes), collectively termed isoprenoids (Fig. 1). These compounds serve a multitude of functions in plants, which include roles as defense compounds (Table 4).

**Table 4.** Microbes and their identified effective terpenoid weapons in Arabidopsis

Species	Type	Sensitive toward	Relevant mutants
*Pseudomonas syringae* pv. *tomato*	Bacterium	(E)-β-caryophyllene; α- and β-pinene	*tps21*; *ggr1*
*Pythium irregulare*	Oomycete	DMNT	*abds*; *cyp705a1-1*

#### (E)-β-caryophyllene

One particular role of terpenoid compounds is acting as pollinator attractants (Dötterl and Gershenzon 2023). However, at least (E)-β-caryophyllene, which is emitted from the stigma of the Arabidopsis flower, also shows antimicrobial activity (Huang et al. 2012). Mutants of *terpene synthase 21* (*tps21*) no longer produce (E)-β-caryophyllene, and the stigmatic tissue of these plants is more heavily colonized by *P. syringae* pv. *tomato* DC3000 (Huang et al. 2012). This results in a reduction of fecundity and seed health. Conversely, overproducing (E)-β-caryophyllene by overexpressing *TPS21* reduces bacterial infection, and the bactericidal activity of (E)-β-caryophyllene was also confirmed in vitro (Huang et al. 2012). Thus, among the bouquet of volatiles emitted by Arabidopsis flowers, there are also compounds that provide protection to the plants from pathogens.

#### α- and β-pinene

Two other volatile terpenoids that act in defense are α- and β-pinene. Firstly, these compounds are produced in the aboveground tissue of Arabidopsis in response to the recognition of the bacterial effector AvrRpm1 (Riedlmeier et al. 2017). Emission of α- and β-pinene by these defense-triggered plants is sensed by neighboring plants, which now induce systemic defense responses such as salicylic acid biosynthesis (Riedlmeier et al. 2017). Fascinatingly, this cross-plant meta-systemic response now provides the unharmed plants with heightened protection against *P. syringae*. Moreover, *geranylgeranyl reductase1* (*ggr1*) mutants with lower levels of α- and β-pinene show normal local defense responses against bacterial infection but are defective in systemic acquired resistance activation. Intriguingly, this can be restored by treating with α- and β-pinene (Riedlmeier et al. 2017). Thus, while (E)-β-caryophyllene provides local protection of the emitting tissue, α- and β-pinene act over long distances.

#### Thalianin, arabidin, and DMNT

While volatiles have mostly been identified and studied in aboveground plant tissues, Arabidopsis also produces and exudes terpenoids from its roots. In a remarkable study, Huang and co-workers identified a network of triterpene biosynthetic gene clusters that produce more than 50 different root-specific metabolites associated with terpene metabolism. Of these, thalianin and arabidin stand out with their activity being induced by jasmonic acid signaling and their ability to be exuded from the root (Huang et al. 2019; Bai et al. 2021). Mutating the underlying biosynthetic machinery, *THALIANOL SYNTHASE 1 (THAS1), THALIANOL HYDROXYLASE (THAH), THALIAN-DIOL DESATURASE (THAD),* and *HXXXD-TYPE ACYL-TRANSFERASE FAMILY PROTEIN (THAA2)* gives rise to significant changes in the exuded terpenoid profile, which correlated with an altered root microbiome when plants were grown in a natural soil from the Chang-ping Farm in Beijing. Conversely, in vitro treatment of diverse root-associated bacterial strains isolated from this soil with purified triterpenes demonstrates selective promotion or suppression of specific strains (Huang et al. 2019).

The triterpene arabidin is produced from arabidiol, which can also serve as a precursor to produce the homoterpene (E)-4,8-dimethyl-1,3,7-nonatriene (DMNT) via cleavage by CYP705A1 (Sohrabi et al. 2015). Infection of the root by *P. irregulare* induces JA biosynthesis and DMNT emission from the root. Since DMNT reduces *P. irregulare* oospore germination in vitro, it can be assumed that oospore germination suppression in the soil might be 1 effect of the emission of this volatile. Further, spore formation in the Arabidopsis root was also enhanced in arabidiol and DMNT biosynthesis mutants (*abds* and *cyp705a1-1*), further supporting a role for these compounds in the defense against *P. irregulare* (Sohrabi et al. 2015).

Thus, just as volatile organic compounds produced in the aboveground parts of the plant act as messengers for intra- and inter-species communication, a similar role may be performed by the compounds exuded from roots. Hopefully, more evidence for this will be provided in the near future, as root exudation of volatile compounds represents an intriguing area of chemical warfare in plants of which we know very little.

Outstanding questions box We now have tools to bring Arabidopsis chemical defense research to the next generation. The examples highlighted here emphasize several unanswered questions.

**How do defense systems work in their natural conditions?**

Plants do not normally grow/live in laboratory conditions. The time is ripe to start probing if chemical defenses hold true in complex, near-native conditions.

**How do chemical activation systems such as myrosinases work?**

With the well-defined activation of GSL in hand, we can probe other BGLUs to identify novel activation systems.

**Are defense compounds sensed and integrated in immune responses?**

Weapons are dangerous to deploy, and we know little of how plants sense and integrate production when needed.

**How is specialized metabolism organized at the cellular level?**

The organization of factories that make chemicals is relatively unknown and may reveal novel regulatory mechanisms.

## Future directions

From the work presented here, it is clear that while our knowledge of the Arabidopsis chemical defense systems is substantial, there are still quite a number of things yet to be discovered (see Outstanding questions box). The ability to easily assess damage caused by pathogens and the underlying genetic responses in this plant makes it deserving of the spot as a top model in plant biology. However, as in all science, asking questions only raises a million more, and this is indeed also the case for work related to Arabidopsis chemical defenses. Below, we have identified some of the future directions where we see potential for Arabidopsis in further advancing our holistic understanding of plant defense mechanisms. This is by far not exhaustive but merely represents the authors' favorite point of view for intriguing questions on the rise.

### How do defense systems work in their natural conditions?

The ease of Arabidopsis growth has empowered researchers to isolate and focus on the investigation of individual variables in defense mechanisms. This powerful approach has given us a comprehensive genetic understanding of the underlying, individual components. However, in many cases it remains to be investigated how and if these systems work under multi-dimensional, complex conditions found in nature, and, importantly, how the different compounds interact and work together (Kerwin et al. 2015). The ease of working with individual knockout mutations as well as the rise of modern genetic manipulation has made it feasible to study the individual contributions of the different compounds, but in nature they do not act in isolation. Accordingly, it is becoming increasingly clearer that we need to establish how these compounds cross-regulate each other and what the cumulative effects of different combinations of these compounds on the microbes are. Now that the majority of the individual components are understood, it is time to move on to a more complex and comprehensive description of the chemical defense systems. Perhaps of similar urgency, it is time to test whether the models obtained with plants grown under artificial, sterile conditions hold up if the plants are grown in their natural, biologically active environments. One important aspect of this is that we know very little about how (and where) immunity mechanisms are deployed underground. This is particularly important, since roots are continuously surrounded by a large community of soil-dwelling microbes—some beneficial, some pathogenic—that make up the root-associated microbiota. All of these microbes have their own sensitivities and resistances, which requires that plants continuously monitor—and also manipulate—these communities to shape them to their benefit. This ability to interact and manipulate the surrounding microbiota is, in all likelihood, a key function of many of the defense compounds discussed here. The rise of new assay mechanisms such as synthetic community establishment for microbes (Northen et al. 2024), as well as the deployment of natural soils, are promising directions for better underground understanding of Arabidopsis defenses.

### How do chemical activation systems such as myrosinases work?

Myrosinases are assumed to be essential detonators for GSL function. Yet, almost all work on a molecular level is done on TGG1 and 2, and PEN2. Maybe even more remarkably, those 3 myrosinases have—more or less—all been studied in the context of IGSLs, while the enzymes involved in the activation of AGSLs are still underexplored. Similarly, while some work has been done to understand the intracellular localization of PYK10 and its role in microbiota assembly, its substrates and mode of action are still unknown. Considering the diversity of the BGLU gene family and lack of enzymatic assessment, there are likely many more myrosinases hidden within the large BGLU family, and with the modern tools available in the Arabidopsis community, it is now feasible to systematically test if they have any specificity to distinct GSLs or GSL classes. Moreover, activation could depend on spatial coincidence, which can be regulated both with spatially distinct expression patterns as well as targeted protein localization, and which needs to be investigated with cellular resolution.

### Are defense compounds sensed and integrated in immune responses?

Defense chemicals are often a direct output of activated immunity responses, yet there are studies showing that the defense compounds themselves also serve as signaling molecules—both to bolster the immune signal and also to provide feedback into developmental pathways (Clay et al. 2009; Kerwin et al. 2011; Kim et al. 2015; Katz and Chamovitz 2017; Malinovsky et al. 2017; Katz et al. 2020). Numerous receptors and NLRs with unknown functions exist, which makes it possible that such sensing systems occur in plants to fine-tune and wind down responses when no longer needed. Indeed, GSL breakdown products have recently been identified as electrophile signaling compounds responsible for long-distance signaling within leaves (Gao et al. 2023). In line with this, certain GSLs can only accumulate to distinct levels before being biochemically modified into other versions (Burow et al. 2015; Jensen et al. 2015). This cannot happen if the levels are not sensed first. Thus, at least for GSLs, unknown sensing mechanisms must exist. Further studies aimed at profiling plants or situations where specialized metabolites must be sensed will shed light on these mechanisms. Here, Arabidopsis and the GSL system are again excellent model for this.

### How is specialized metabolism organized at the cellular level?

The highly labile intermediates of specialized metabolisms, as well as the tight connection to pathways such as amino acid synthesis, suggest that formation of these compounds is tightly controlled at the subcellular level. Moreover, many pathways are compartmentalized between organelles. In the late 1970s, the formation of supermolecular complexes, so-called metabolons, was proposed to allow tight metabolic flux between enzymes (Welch 1978; Srere 1984, 1985; Andersen 2012). Several prominent examples of this exist, in particular in the phenylpropanoid pathway, as well as within GSL formation (Jørgensen et al. 2005; Andersen 2012; Mucha et al. 2019). Nonetheless, we are only beginning to understand how these pathways are organized (Obata 2019). One additional observation is that such pathways can be split between different cells, as exemplified recently in *Catharanthus roseus* (Madagascar periwinkle), where essential parts of the biosynthetic machinery underlying the formation of vinblastine are split across multiple cell types (Kulagina et al. 2022). It is likely that the rise of tools with high spatial resolution, such as single-cell analysis, spatial transcriptomics, and high-resolution live imaging, will reveal insights into such things in Arabidopsis as well.

## Data Availability

No orignal data is produced in this work. But the authors are happy to discuss details upon contact.

## Dive Curated Terms

The following phenotypic, genotypic, and functional terms are of significance to the work described in this paper:

BGLU42 Gramene: AT5G36890
BGLU42 Araport: AT5G36890

MYB51 Gramene: AT1G18570
MYB51 Araport: AT1G18570

TGG1 Gramene: AT5G26000
TGG1 Araport: AT5G26000

MYB28 Gramene: AT5G61420
MYB28 Araport: AT5G61420

MYB72 Gramene: AT1G56160
MYB72 Araport: AT1G56160

BGLU21 Gramene: AT1G66270
BGLU21 Araport: AT1G66270

BGLU22 Gramene: AT1G66280
BGLU22 Araport: AT1G66280

MYB15 Gramene: AT3G23250
MYB15 Araport: AT3G23250

PAD1 Gramene: AT3G51260
PAD1 Araport: AT3G51260

PAD2 Gramene: AT5G66140
PAD2 Araport: AT5G66140

pen2 Gramene: AT2G44490
pen2 Araport: AT2G44490

WRKY33 Gramene: AT2G38470
WRKY33 Araport: AT2G38470

PYK10 Gramene: AT3G09260
PYK10 Araport: AT3G09260

PDR12 Gramene: AT1G15520
PDR12 Araport: AT1G15520

PAD3 Gramene: AT3G26830
PAD3 Araport: AT3G26830
